# Innovation and Inequality: A Medical Student Perspective. Comment on "The Present and Future Applications of Technology in Adapting Medical Education Amidst the COVID-19 Pandemic"

**DOI:** 10.2196/26790

**Published:** 2021-10-04

**Authors:** Myat Pan, Myat San

**Affiliations:** 1 Cardiff University School of Medicine Cardiff United Kingdom; 2 University of Oxford Medical School Oxford United Kingdom

**Keywords:** medical education, technology, coronavirus, medical students, COVID-19, pandemic, online lecture, virtual reality, education

We read with great interest a *JMIR Medical Education* article from Remtulla [1] on the merits of using technology in medical education amidst the COVID-19 pandemic. The article resonated greatly with our experiences as fifth-year medical students during the pandemic, which has created an opportunity to innovate and redefine medical education in an age of technology.

First, the author suggests online lectures may become a permanent alteration. We find Zoom or Microsoft Teams convenient and practical to use for clinical teaching, while our colleagues agree that concentration is better in our own rooms than in lecture theaters. Moreover, it is easier to see and annotate clinical pictures on laptop screens. Thus, we support virtual clinical teaching, particularly in specialties such as radiology and dermatology where it has been the most useful. However, online lectures may risk losing rapport between educators and students [2]. This is especially true for prerecorded lectures, which may reduce engagement if students cannot ask questions for clarification or prompt discussions. There may also be health consequences from increased screen time [2]. Therefore, we recommend synchronous online learning, where students engage in virtual classes and live webinars in real time with their lecturers and peers, which can complement face-to-face teaching once the pandemic resolves.

Secondly, we commend the author for highlighting the importance of incorporating telemedicine into medical education. On placement, we witnessed the benefits of virtual clinics for frail patients such as in old-age psychiatry or movement disorder clinics where consultations can be done from their own homes. Doctors can also accommodate more students to observe and learn within a conference call, whereas space is a limiting factor with physical consultations. The pandemic demonstrated our unpreparedness, as students, for history-taking and examination in video calls, despite the difficulty of them for even experienced professionals. Undoubtedly, the use of telemedicine will increase post–COVID-19, which makes it an urgent case for telemedicine to be taught early on in medical school. Studies from the United States show high student satisfaction and engagement where telemedicine is a component of the medical curriculum [3]. Therefore, we would appreciate more virtual history-taking and examination skills teaching at medical school. This can perhaps take place during community placements as general practitioners already utilize telemedicine day to day in their practice. Medical schools can also offer student-selected modules within telemedicine in preclinical years for early exposure.

Finally, while we value the author’s suggestions for medical education going forward, we are concerned about the limits of technology in widening participation [4]. We want to ask the author’s and readership’s opinions on widening access for students who may not benefit from a digitalized education such as those from socially disadvantaged backgrounds or people with disabilities. However, we acknowledge that online teaching holds the potential to even increase equity for students by enabling flexible working patterns and participation from different geographical locations. In our experience, the pandemic has shown many merits in utilizing technological advances for medical education, but much work is still yet to be achieved to provide an equitable one.
